# Cellular Renewal and Improvement of Local Cell Effector Activity in Peritoneal Cavity in Response to Infectious Stimuli

**DOI:** 10.1371/journal.pone.0022141

**Published:** 2011-07-22

**Authors:** Alexandra dos Anjos Cassado, José Antônio Tavares de Albuquerque, Luiz Roberto Sardinha, Carina de Lima Buzzo, Lucas Faustino, Rogério Nascimento, Eliver Eid Bou Ghosn, Maria Regina D'Império Lima, Jose Maria Mosig Alvarez, Karina Ramalho Bortoluci

**Affiliations:** 1 Departamento de Ciências Biológicas, Campus Diadema e Centro de Terapia Celular e Molecular (CTC-Mol), Universidade Federal de São Paulo, Diadema, São Paulo, Brasil; 2 Departamento de Imunologia, Instituto de Ciências Biomédicas, Universidade de São Paulo, São Paulo, Brasil; 3 Instituto Israelita de Ensino e Pesquisa Albert Einstein, São Paulo, Brasil; 4 Department of Genetics, Stanford University School of Medicine, Stanford, California, United States of America; Fundação Oswaldo Cruz, Brazil

## Abstract

The peritoneal cavity (PerC) is a singular compartment where many cell populations reside and interact. Despite the widely adopted experimental approach of intraperitoneal (i.p.) inoculation, little is known about the behavior of the different cell populations within the PerC. To evaluate the dynamics of peritoneal macrophage (MØ) subsets, namely small peritoneal MØ (SPM) and large peritoneal MØ (LPM), in response to infectious stimuli, C57BL/6 mice were injected i.p. with zymosan or *Trypanosoma cruzi*. These conditions resulted in the marked modification of the PerC myelo-monocytic compartment characterized by the disappearance of LPM and the accumulation of SPM and monocytes. In parallel, adherent cells isolated from stimulated PerC displayed reduced staining for β-galactosidase, a biomarker for senescence. Further, the adherent cells showed increased nitric oxide (NO) and higher frequency of IL-12-producing cells in response to subsequent LPS and IFN-γ stimulation. Among myelo-monocytic cells, SPM rather than LPM or monocytes, appear to be the central effectors of the activated PerC; they display higher phagocytic activity and are the main source of IL-12. Thus, our data provide a first demonstration of the consequences of the dynamics between peritoneal MØ subpopulations by showing that substitution of LPM by a robust SPM and monocytes in response to infectious stimuli greatly improves PerC effector activity.

## Introduction

The mouse peritoneal cavity (PerC) is a unique environment characterized by the co-habitation and interaction of many cell types, like within secondary lymphoid organs. In steady state conditions, the majority of peritoneal cells (PC) are macrophages (MØ) and B-1 cells; conventional B-2 cells, T cells, natural killer (NK) cells, dendritic cells (DC) and granulocytes (mostly eosinophils) are also present [1], [2]. In addition to the complexity of the cellular composition in the PerC, we have recently demonstrated that peritoneal MØ comprise two distinct subsets named small peritoneal MØ (SPM) and large peritoneal MØ (LPM) [1]. SPM and LPM differentially express a variety of surface markers, including pattern recognition receptors (PRR) and proteins involved in antigen presentation and cell migration [1]. Importantly, SPM and LPM appear to have distinct responses to LPS stimulation *in vitro* and *in vivo*, indicating that these peritoneal MØ cannot be considered a single population.

As a heterogeneous population, MØ play a wide range of roles in the immune system; they are involved in the removal of apoptotic cells and cellular debris under steady state conditions [3], microorganism ingestion and elimination [4], [5], antigen presentation and consequent T cell activation [6], [7], resolution of inflammatory processes, angiogenesis and tissue repair [8]. The functionality of MØ subsets is influenced by factors that include their maturation state and activation profile.

The maturation state influences the functional behavior of MØ; their performance is particularly compromised as they age [9]–[14]. Age-related debilitation is reflected in activities associated with MØ microbicidal function, including impairment in phagocytic ability and in the production of inflammatory cytokines, chemokines and free radicals; additionally, expression of MHCII molecules is diminished, which may contribute to poor CD4^+^ T cell responses. Morphologically, senescent MØ adopt a flattened, enlarged morphology and exhibit molecular markers like the senescence-associated β-galactosidase (β-gal) [15]. Accumulation of senescent MØ can compromise the tissues architecture and fitness [9]–[13].

MØ subsets can assume distinct functional profiles depending on environmental stimuli. Classically-activated MØ, also known as M1 MØ, are generated in the presence of LPS and IFN-γ; they exhibit high microbicidal ability and secrete pro-inflammatory cytokines [16]–[19]. Conversely, alternative or M2 MØ are generated in the presence of immune-complexes (IC) or Th2 cytokines and appear to be responsible for the control of inflammatory processes and tissue repair. They have also been implicated in managing helminth infections [20], [21].

It is reasonable to assume that in each tissue, MØ develop particular characteristics and become capable of unique functions. For instance, osteoclasts have the unique capacity for bone remodeling, alveolar MØ possess the ability for self/non self discrimination and lamina propria MØ exhibit high microbicidal and phagocytic capacities coupled with low production of pro-inflammatory cytokines [5]. However, distinct MØ subpopulations have been found in the same environment [1], [5], and little is known about their relative contribution to tissue/organ function. Since the majority of studies focus on *in vitro* MØ activities, many questions about MØ behavior and function inside tissue microenvironments remain unsolved. In the present work, we examined the dynamics between SPM and LPM within the PerC in response to exposure to zymosan, an insoluble carbohydrate from the cell wall of yeast, and live *Trypanosoma cruzi* (*T. cruzi*) parasites.

## Materials and Methods

### Mice and parasites

Six- to eight-week-old female C57BL/6 mice were bred in our animal facilities at the University of São Paulo under standard pathogen-free conditions. All animal studies and protocols were approved by the University of São Paulo Committee for the Use and Care of Animals (Number 109/07). *T. cruzi* trypomastigotes of the Sylvio-X10/4 strain were purified from LLC-MK_2_ cells (a monkey epithelial cell line derivative from American Type Culture Collection (ATCC CCL-7), USA).

### Peritoneal cell suspensions

Peritoneal cells (PC) were harvested by flushing the PerC of mice, as previously described [22]. Total PC and purified peritoneal MØ subsets obtained after cell-sorting were cultured in RPMI 1640 (Sigma-Aldrich, St. Louis, MO) supplemented with L-glutamine (2 mM) and 3% heat-inactivated fetal calf serum (FCS) at 37°C and 5% CO_2_. All supplements were purchased from Life Technologies (Rockville, MD).

### *In vivo* stimulation

For *in vivo* stimulation assays, mice were injected i.p. with zymosan particles (1 mg/mouse) or *T. cruzi* trypomastigotes of the Sylvio-X10/4 strain (10^6^ parasites/mouse). PC were harvested after 30 min or 48 h.

### *In vitro* stimulation

Total PC (10^6^ cells) from naive or *in vivo*-inoculated mice were cultured for 10 h in the presence of brefeldin A (BD Biosciences, Heidelberg, Germany) at 37°C and 5% CO_2_. Cells were stimulated with LPS from *Escherichia coli* (Sigma-Aldrich) (1 µg/mL) and rIFN-γ (5 ng/mL) (BD Biosciences). Purified MØ subsets (2×10^5^) were cultured in 8-well chambers for 12 h at 37°C and 5% CO_2_. Next, cells were fixed and labeled according to the InstantProv KIT (NewProv, Brazil) instructions.

### Flow cytometry

Cells were phenotypically analyzed by flow cytometry as previously described [23], with some modifications. Briefly, cell suspensions were stained for 30 min with a combination of following biotin- or fluorochromes- conjugated monoclonal antibodies (mAbs): fluorescein-isothiocyanate (FITC)-labeled anti- F4/80 (CI:A3-1) (Serotec, Düsseldorf, Germany); phycoerythrin (PE)-labeled anti-CD80 (16-10A1), anti-CD86 (GL1), anti-CD40 (3/23), anti-CD11b (M1/70), anti–IA^b^ (AF6-120.1) or anti-LY6C (AL-21) (BD Biosciences); allophycocyanin (APC)-labeled anti-CD19 (1D3) (BD Biosciences), PerCP- conjugated anti-CD11c (HL3) (BD Biosciences) and biotin-conjugated anti–IA^b^ (AF6-120.1), anti-TLR-4 (MTS510), anti-DC-Sign (5H10/CIRE) (BD Biosciences) or anti-Dectin-1 (2A11) (Serotec). For the detection of biotinylated antibodies, streptavidin-Alexa Fluor 405 (BD Biosciences) was used. For the detection of intracellular IL-12, PC were stained using the combination of FITC-labeled anti-F4/80, APC-labeled anti-CD19, PerCP- labeled anti-CD11c (HL3) and biotin-conjugated anti–IA^b^ (AF6-120.1) as described above, fixed with Cytofix (BD Biosciences) for 20 min, and then incubated with PE-labeled polyclonal anti-mouse antibodies against IL-12p70 (C15.6) (BD Biosciences) for 30 min in Cytoperm buffer (BD Biosciences) [24]. For all experiments, 2×10^5^ events were acquired. All acquisitions were performed using a 5-color staining combination and acquired on FACSCalibur or FACSCanto II (BD Biosciences). Analysis was carried out with FlowJo software (Tree Star). Fluorescence-minus-one (FMO) controls [23], [25] were used to distinguish positive staining from autofluorescent cells. Peritoneal MØ subsets were sorted using a FACSVantage (BD Biosciences), according to the parameters described in Fig. S1.

### Adherent-cell culture

PC obtained from control and injected animals (6×10^5^ cells) were incubated in 96-well plates (Costar) for 4 h at 37°C and 5% CO_2_. The non-adherent cells were removed by vigorous washes with RPMI medium.

### Nitric Oxide measurement

Adherent PC from control and injected mice were cultured with medium, LPS from *Escherichia coli* (Sigma-Aldrich) (1 µg/mL) or LPS plus rIFN-γ (5 ng/mL) (BD Biosciences); supernatants were harvested after 48 h. Culture supernatants were assayed for NO by the Griess reaction. Briefly, 50 µl of supernatant was incubated with 50 µl of Griess reagent for 5 min at room temperature. Nitrite concentration was determined by measuring the optical density at 550 nm in reference to a standard sodium nitrite solution.

### β-Galactosidase (β-Gal) staining

β-Gal staining was performed following the conventional protocol for senescence associated (SA)-β-gal staining [15]. Briefly, adherent-PC from naive or injected mice were fixed in 2% formaldehyde/0.2% glutaraldehyde for 5 min at room temperature, washed with PBS and then incubated overnight at 37°C with β-Gal staining solution at pH 6.0. β-Gal solution was composed of 1 mg/mL of X-gal (5-bromo-4-chloro-3 indolyl–galactopiranoside); 40 mM of citric acid/sodium phosphate, pH 6.0; 150 mM sodium chloride; 2 mM magnesium chloride; 5 mM potassium ferrocyanide; and 5 mM potassium ferricyanide. Cells were observed by phase contrast on a Nikon TS100 microscope and photographed at 40× magnification.

### Statistical analysis

Statistical analyses were performed using the unpaired analysis of variance ANOVA (Tukey Test) test. Differences between two groups were considered significant when p<0.05.

## Results

### F4/80 and MHCII expression distinguishes LPM and SPM

**Figure 1:**
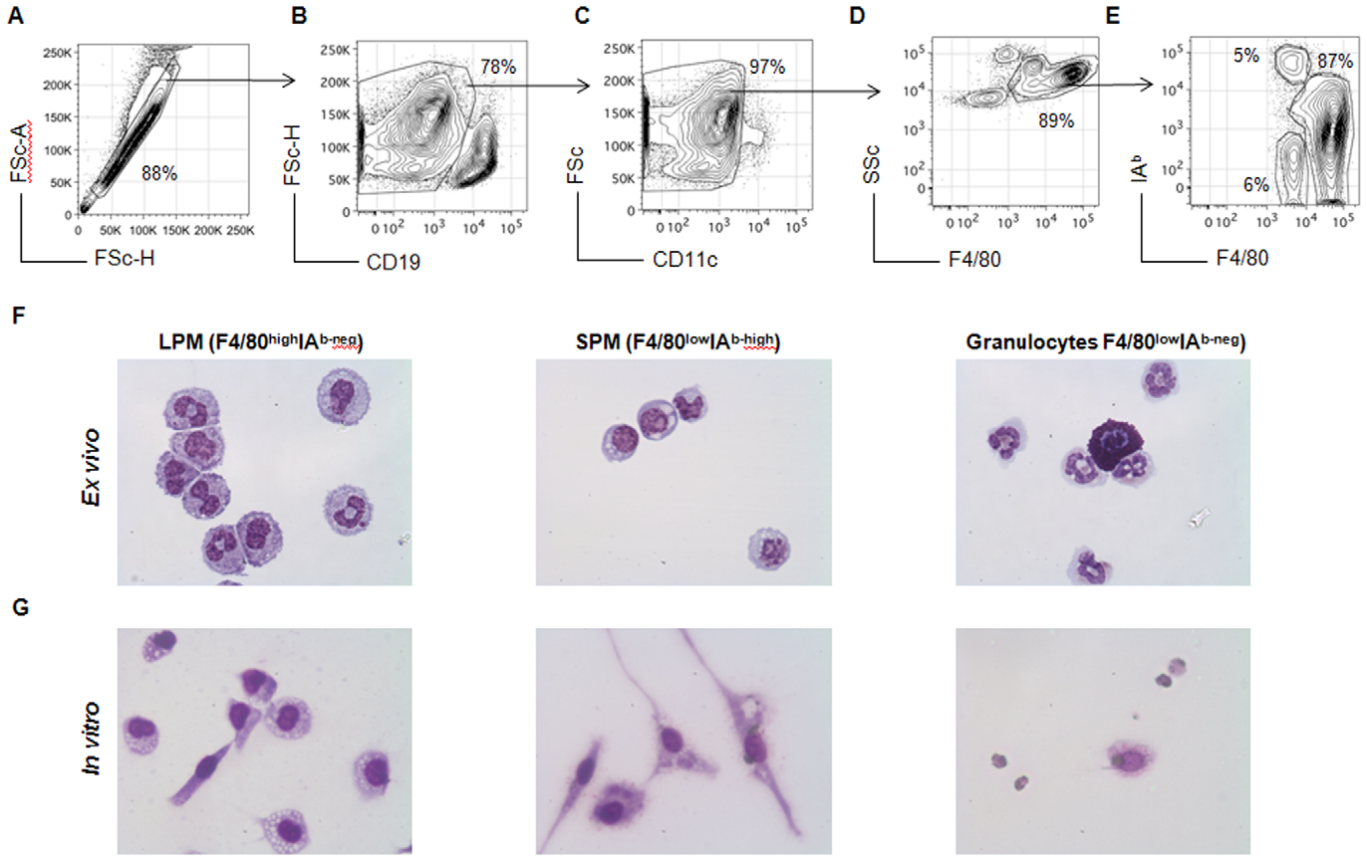
Identification of resident peritoneal MØ subsets. PC from C57BL/6 were harvested and stained with fluorochrome-labeled antibodies directed against F4/80, CD19, CD11c and IA^b^ for flow cytometry analysis. (**A**) Doublet cells were excluded according to forward scatter profiles (FSC-A and FSC-H). Subsequently, (**B**) CD19^high^ cells and (**C**) CD11c^high^ cells were also excluded, and (**D**) F4/80^+^ cells were selected. (**E**) F4/80 and IA^b^ expression defined three populations: LPM (F4/80^high^IA^b-neg^), SPM (F4/80^low^IA^b-high^) and granulocytes (F4/80^low^IA^b-neg^). These three subpopulations were purified by cell sorting on a FACS Vantage, and their morphology was evaluated *ex vivo* from cytospin slides (**F**), or after *in vitro* culture in chamber slides for 12 h (**G**). Slides were stained with hematoxylin and eosin (H&E) and analyzed by optical microscopy (40×).

Utilizing a high-dimensional digital FACS with 11-color combination, we recently demonstrated that peritoneal MØ comprises two distinct subpopulations, namely large (LPM) and small peritoneal MØ (SPM); these subpopulations have phenotypes and functions distinct from monocytes and DC [1]. Here, we demonstrate that these two subpopulations can also be identified using a 4-color flow cytometry staining panel. First, doublet cells (Fig. 1A) and CD19^high^ cells (Fig. 1B) were excluded. Next, CD11c^high^ cells were excluded to eliminate remaining DC (Fig. 1C). The Side Scatter (SSC) profile and F4/80 expression revealed two distinct F4/80^+^ subpopulations among CD19^neg/verylow^CD11c^neg/verylow^ cells (Fig. 1D). In addition, analysis of F4/80^+^ cells according to MHCII (IA^b^) expression defined three distinct subpopulations (Fig. 1E). Sorting of each population revealed that F4/80^high^IA^b-neg^, F4/80^low^IA^b-high^ and F4/80^low^IA^b-neg^ correspond, respectively, to LPM, SPM and granulocytes (mostly eosinophils) (Fig. 1F). After 12 h in culture, SPM acquire a polarized morphology whereas LPM exhibit prominent vacuolization. Granulocytes appear to die after *in vitro* culture (Fig. 1G). As granulocytes present a typical SSC profile with no MHCII expression (Fig. 1F and Fig. S1A), F4/80^+^ cells were gated to exclude F4/80^low^SSC^high^ cells to establish the efficient identification of peritoneal MØ subsets (Fig. S1B). Using a 5-color flow cytometry staining panel, we observed that SPM and LPM displayed the typical expression of surface markers, as previously demonstrated [1] (Table 1). In addition to the previous published phenotypic features [1], higher expression of the endocytic receptors Dectin-1 and DC-Sign (dendritic cell-specific intercellular adhesion molecule-grabbing nonintegrin, CD209) was observed in SPM as compared to LPM (Table 1).

**Table 1:** Phenotypic analysis of resident peritoneal MØ subsets.

Surface molecule	LPM	SPM
F4/80	1970	90
CD11b	1700	30
IA^b^ (MHC-II)	= FMO	3100
CD80	140	100
CD86	250	10
CD40	270	230
TLR4	150	80
DC-Sign	10	100
Dectin-1	840	1600
Ly6C	= FMO	= FMO

SPM and LPM were gated as shown in Figure 1 and the median fluorescence intensity (MFI) of F4/80, CD11b, IA^b^, CD80, CD86, CD40, LY6C, DC-SIGN, Dectin-1 and TLR4 was analyzed by flow cytometry. All acquisitions were performed using a 5-color staining combination. The value for the expression of each surface marker was obtained by subtracting the MFI of the fluorescence-minus-one control (FMO) from the MFI of the whole stained cell population [23], [25], as described in the Materials and Methods (M&M) section. Data are representative of more than 3 independent experiments.

### Zymosan and *T. cruzi* exposure alter the balance between SPM and LPM

**Figure 2:**
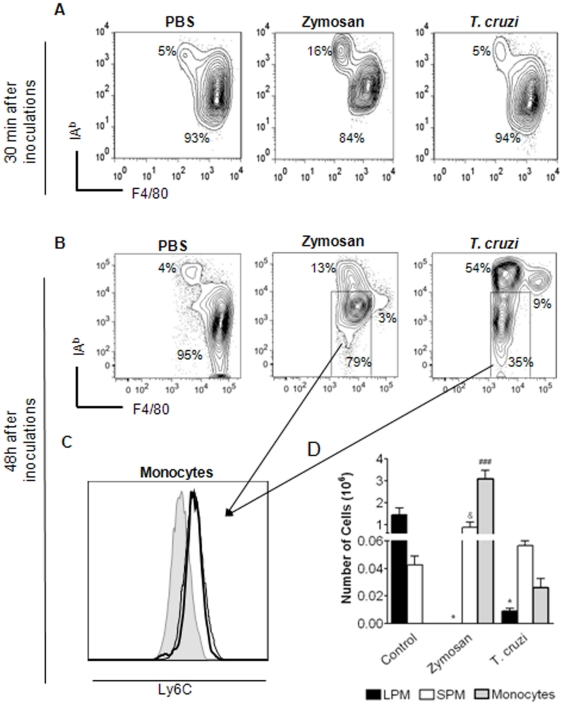
Zymosan and *T. cruzi* injection alters the MØ compartment of PerC. C57BL/6 mice were injected i.p. with zymosan (1 mg/mouse) or *T. cruzi* (10^6^ parasites/mouse) and at 30 min (**A**) or 48 h (**B**) after stimulation, PC from naive and injected mice were harvested and stained as described in M&M. Sequential gates were made as shown in Fig. S1. Plots show the frequencies of each subpopulation. (**C**) F4/80^low^MHCII^int^ cells present within PerC 48 h after injections were evaluated according the expression of Ly6C. Gray lines represent FMO [23], [25], the black lines show F4/80^low^MHCII^int^ cells from zymosan- (hairline) or *T. cruzi*-(bold line)-exposed PerC. (**D**) Total numbers of SPM, LPM and monocytes 48 h after zymosan or *T. cruzi* exposure (within MØ gate) are shown in panel. Data are representative of more than 3 independent experiments.

The PerC is a dynamic compartment that selectively attracts immune cells, particularly in response to injury, which can result in major alteration in their cellular composition. To evaluate the dynamics of LPM and SPM in response to infectious stimuli, C57BL/6 mice were injected i.p. with zymosan or live *T. cruzi* trypomastigotes, and PC were collected after 30 min or 48 h. Minor alterations in cell numbers and composition were observed at early time points after i.p. injections (Fig. 2A and data not shown); however, an increase in the frequency of SPM in response to zymosan was observed (Fig. 2A). Conversely, 48 h after zymosan and *T. cruzi* exposure there was a clear alteration in the myelo-monocytic cell compartment of the PerC, characterized by a dramatic decrease in the numbers of LPM coupled with increased frequency of SPM (Fig. 2B and 2D). Moreover, a massive influx of cells with a monocytic phenotype (F4/80^low^MHCII^int^Ly-6C^+^) was noted (Fig. 2B, 2C and 2D). Since both SPM and monocytes that infiltrate PerC showed same levels of F4/80, they were distinguished by Ly-6C and MHCII expression. SPM displayed high levels of MHCII and Ly-6C expression was found only on monocytes (Fig. 2B, 2C and Table 1). The enrichment of monocytes was particularly observed after zymosan exposure when F4/80^low^MHCII^int^Ly-6C^+^ cells became the dominant population in the PerC (Fig. 2D). Interestingly, 48 h after *T. cruzi* stimulation, cells displaying the phenotypic features of SPM (F4/80^low^MHCII^hi^Ly-6C^−^) become the major peritoneal myeloid cell population (Fig. 2B and 2D). Despite the fact that *T. cruzi* and zymosan induce distinct modifications in the PerC, both stimuli led to SPM dominancy over LPM.

### Modification in the myelo-monocytic compartment results in the PerC cellular renewal and improvement of nitric oxide and IL-12 responses to a subsequent LPS stimulation

**Figure 3:**
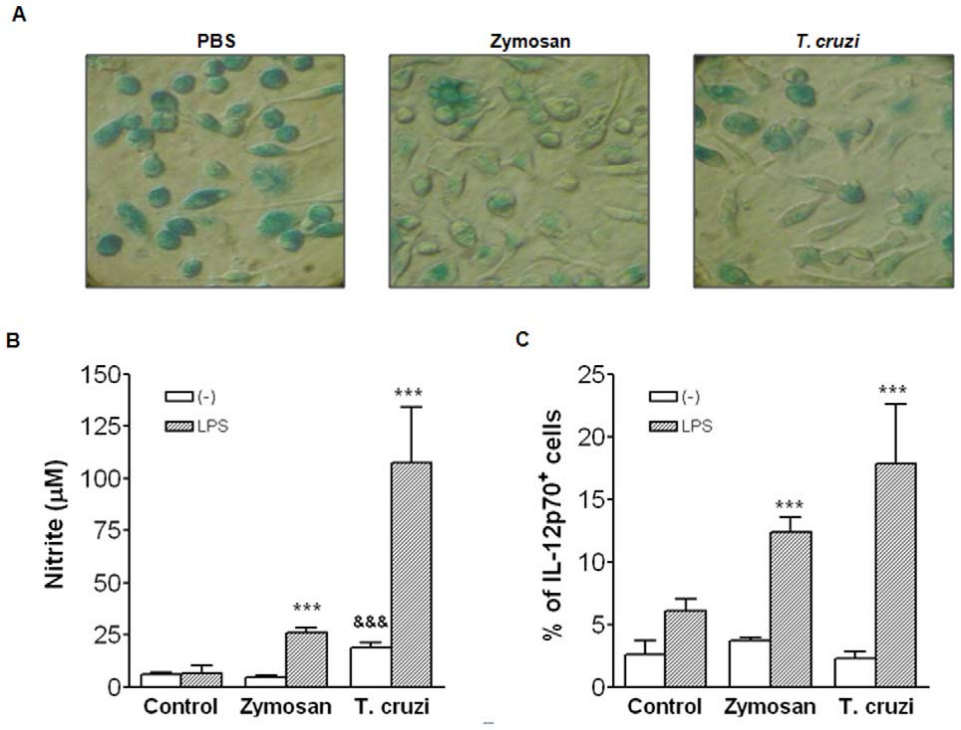
*Zymosan and* T. cruzi *inoculation* leads to PerC cellular renewal and improves local cell effector activities. C57BL/6 mice were injected i.p. with zymosan (1 mg/mouse) or *T. cruzi* (10^6^ parasites/mouse) and 48 h later PC from naive and injected mice were harvested and cultured for 4 h. Non-adherent cells were removed by sequential washes. (**A**) β-Gal staining was performed as described in M&M. Slides were analyzed by optical microscopy (40×) and pictures were captured by Sony Camera (zoon 3.0). (**B and C**) Adherent PCs cells from 48 h-exposed mice and controls were stimulated *in vitro* with media or LPS (1 µg/mL). (**B**) Nitrite concentrations in culture supernatants were determined by the *Griess* reaction after 48 h of *in vitro* culture. Numbers represent the mean ± SD of triplicate samples. ***p<0.001 in relation to non-stimulated group and &&& *p*<0.001 when compared to the control group. (**C**) Frequencies of IL-12-producing F4/80^+^ cells were evaluated by intracellular staining as described in M&M. Bars indicate the standard deviation (SD). *** *p*<0.001 when compared to the control group. Data are representative of more than 3 independent experiments.

Given that peritoneal myelo-monocytic cell composition is profoundly altered 48 h after zymosan and *T. cruzi* exposure, we decided to examine the impact of this alteration for the renewal and response of PC to a subsequent LPS stimulation. As it is well established that senescent cells can accumulate in tissues compromising tissue fitness [9]–[13], [15], we compared β-Gal expression, a marker of cell senescence [15], in PerC from control and 48 h-zymosan or *T. cruzi*-exposed mice. For the enrichment of cells from myelo-monocytic lineage (LPM and SPM within control PerC; SPM and monocytes within stimulated PerC), only adherent cells from PerC were analyzed (a cell population containing 98% of MØ and/or monocytes – data not shown). While a great majority of adherent PC from control mice was positive for β-Gal, a significant reduction in the staining was observed in PC from mice exposed to zymosan and *T. cruzi* (Fig. 3A). Moreover, the frequency of β-Gal-positive cells in control mice correlated with the frequency of LPM present among peritoneal MØ (Fig. 1 and Fig. 2); this finding suggests that the majority of LPM is senescent. In addition, adherent PC from control mice were unable to secrete nitric oxide (NO), a potent microbicidal molecule, even after subsequent LPS stimulation (Fig. 3B). Although adherent PC from *T. cruzi*-exposed mice were able to secrete NO in the absence of LPS, high levels of NO were found in LPS-stimulated PC from both zymosan- and *T. cruzi*-exposed mice (Fig. 3B). As IL-12 is involved in the ability of MØ to secrete NO in response to LPS and IFN-γ [26]–[30], we next examined the frequency of IL-12-producing cells. After *in vitro* LPS stimulation, two to three times higher frequencies of IL-12-producing cells were found within myelo-monocytic cells from mice exposed to zymosan and *T. cruzi* in comparison to the untreated control mice (Fig. 3C).

### Peritoneal myelo-monocytic cell subsets are differentially activated by exogenous stimuli

**Figure 4:**
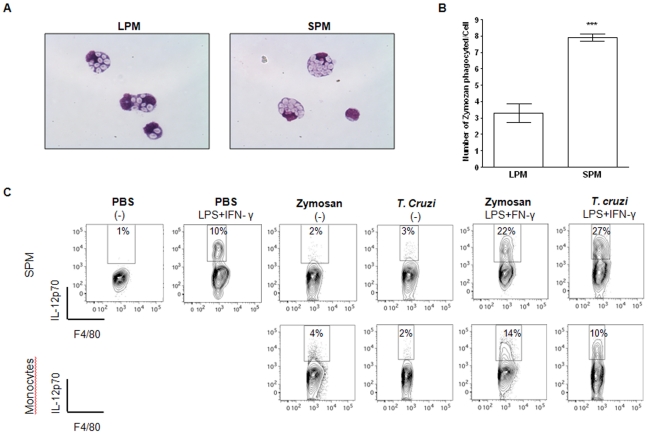
SPM are more responsive than LPM and monocytes to infectious stimuli. C57BL/6 mice were injected i.p. with zymosan (1 mg/mouse) or *T. cruzi* (10^6^ parasites/mouse) and PCs were harvested 30 min or 48 h after stimulation. (**A**) SPM and LPM from C57BL/6 zymosan-exposed mice were FACS-sorted 30 min after injection and the presence of internalized zymosan particles was observed by optical microscopy. Slides were made with 10^5^ cells from purified MØ subsets, stained with H&E and analyzed by optical microscopy (40×). (**B**) Numbers represent the mean ±SD of internalized zymosan particles per cell in each MØ subset. *** *p*<0.001 when compared to the LPM group. (**C**) PC from control or 48 h-exposed mice were cultured for 6 h in the presence of brefeldin A with or without LPS (1 µg/ml) plus rIFN-γ (5 ng/ml). Titles above plots indicate ***in vivo*** - *in vitro* stimulations. Values inside gates represent the frequencies of IL-12-producing cells in each subpopulation. Data are representative of more than 3 independent experiments.

Next, we evaluated the individual contribution of myelo-monocytic cell populations to the improvement of inflammatory responses found in zymosan or *T. cruzi*-stimulated PerC. At an early time point (30 min) after zymosan or *T. cruzi* exposure, LPM and SPM were still the dominant myelo-monocytic cell populations present in the PerC (Fig. 2A). At this time point, higher numbers of zymosan particles were found inside SPM (Fig. 4A and 4B). Forty-eight hours after zymosan or *T. cruzi* exposure, the frequency of SPM spontaneously producing IL-12 was similar to untreated controls. However, when stimulated with LPS plus IFN-γ *in vitro* we observed two to three times more IL-12-producing SPM from zymosan- or *T. cruzi*-treated mice compared to PBS-injected controls (Fig. 4C). Importantly, the IL-12 response to LPS plus IFN-γ was twice as high in SPM from injected mice than in the respective infiltrating monocytes (Fig. 4C). These data indicate that modifications in the peritoneal myelo-monocytic compartment in response to infectious stimuli include the emergence of a robust SPM cell population.

## Discussion

MØ represent a heterogeneous cell population that resides in virtually all tissues of the body where they are able to assume a wide range of immune functions. Depending on their activation profile, these cells have been implicated in pathogen control and the initiation of inflammatory processes, or, conversely, in the regulation of these processes followed by restoration of tissue homeostasis [17]–. It is currently accepted that tissue-derived factors influence MØ activation profiles. However, distinct MØ subpopulations have been found in the same environment [1], [5]. Consistent with this, we have recently described the coexistence of large (LPM) and small (SPM) peritoneal MØ in the PerC [1]. Despite the distinct *ex vivo* and *in vitro* responses to LPS stimulation, the *in vivo* behavior of SPM and LPM remain to be elucidated. Here, we demonstrate that there is a clear alteration in the frequencies of LPM and SPM during inflammatory responses to zymosan and *T. cruzi*. At early time points after zymosan exposure, although LPM remains the major peritoneal MØ subset, the PerC is enriched with SPM. However, 48 h after zymosan and *T. cruzi* stimulation, the cellular composition of the PerC is profoundly altered by the disappearance of LPM and the accumulation of SPM and monocytes. In parallel, cells in the modified PerC appear to lose the expression of the β-galactosamine senescence marker while increase NO secretion and the frequency of IL-12-expressing cells in response to subsequent LPS and IFN-γ stimulation.

Peritoneal MØ have been extensively adopted for experimental models because of their easy enrichment and isolation from the PerC. However, we and others have demonstrated that a variety of cell populations reside in the PerC, including B cells, DC, eosinophils, mast cells, neutrophils, T cells, NK cells, and invariant NK T cells [1], [2], [31]. Under steady state conditions, B-1 cells and MØ compose the majority of PC [1]. We have recently proposed that resident MØ are represented by two subsets, LPM and SPM [1].

The heterogeneity of resident peritoneal myelo-monocytic cells was also reported by Dioszeghy et al. [31] who also defined two distinct populations of MØ-like cells according to the expression of 12/15-lipoxygenase (LOX). Interestingly, these 12/15-LOX^+^ and 12/15-LOX^−^ MØ-like cells display distinct functional and phenotypic properties. The 12/15-LOX^+^ population expresses higher levels of F4/80 and Scavenger Receptor (SR)-A and secrete more IL-10 and G-CSF in response to *Staphylococcus epidermis* cell-free supernatant (SES) than do 12/15-LOX^−^ cells. This F4/80^high^12/15-LOX^+^ subset was defined by authors as resident MØ [31] and are present in the PerC at similar frequencies of LPM in steady state conditions [(1) and (Fig. 1 and 2)]. Because they share functional and phenotypic characteristics, F4/80^high^12/15-LOX^+^ subset and LPM may represent the same cell population.

On the other hand, we believe the F4/80^low^12/15-LOX^−^ cells and SPM are different cell populations. Despite sharing the same CD11b^+^F4/80^low^MHCII^high^ phenotype, the expression of CD11c distinguishes 12/15-LOX^−^ cells and SPM. 12/15-LOX^−^ cells express high levels of the DC marker CD11c [31], whereas SPM was selected among CD11c negative or very low cells (Fig. 1 and [1]). In addition, SPM express lower levels of CD40, CD80 and CD86 costimulatory molecules compared to LPM [(1) and Table 1]. In comparison, 12/15-LOX^−^ cells express higher levels of these same co-stimulatory molecules than its counterpart 12/15-LOX^+^ cells [31].

However, because the expression of CD11c and other surface markers is not homogeneous among DC and MØ subpopulations and the fact that DC and MØ share many of their immune functions [32], [33], we consider the possibility that the discrimination between 12/15-LOX^−^ cells and SPM may not be precise. Therefore, we envision the possibility that these two populations partially overlap and, accordingly, the presence of SPM within F40/80^low^12/15-LOX^−^ cells and vice versa cannot be discarded.

As a dynamic compartment, the PerC recruits effector cells in response to injury. Neutrophil and inflammatory monocyte infiltrates are observed in response to stimuli such as LPS, thyoglicolate, SES or zymosan [1], [31], [34], [35]. A clear alteration in the frequencies of LPM and SPM was observed after zymosan and *T. cruzi* stimulation. After 30 min of exposure, a modest SPM enrichment was observed. However, their numbers were not significantly altered (data not shown). In addition, at late time points after zymosan and *T. cruzi* stimulation, granulocytes, monocytes and SPM appeared to account for the increased cell numbers found in the PerC (Fig. 2B and data not shown). Interestingly, SPM and monocyte enrichment was accompanied by LPM disappearance from the PerC (Fig. 2). We have shown that LPM disappearance in response to thyoglicolate injection cannot be attributed to LPM differentiation into SPM [1]. However, the events underlying LPM disappearance from stimulated PerC that could include cell death or migration remain to be solved.

Many studies have been demonstrated the impact of immunesenescence or age-related immune cell deficiency in MØ functions [9]–[14]. For example, aging impairs production of NO [14] and phagocytic capacity [9], [10], [12] of MØ and reduces their expression of MHC class II [13]. LPM seem to exhibit morphological and functional characteristics described in senescent cells, including large size, prominent vacuolization (Fig. 1), reduced phagocytic capacity (Fig. 4) and low expression of MHCII molecules (Fig. 1) [9]–[14]. Furthermore, these senescent signs were accompanied by defective NO production *in vivo* [1]. In this regard, the senescence could be hypothesized to explain the rapid disappearance of LPM from the PerC after stimulation. In fact, after zymosan and *T. cruzi* exposure, adherent PC exhibited decreased β-Gal staining (Fig. 3) [15], indicating that the loss of LPM correlates to the cell renewal of the PerC. Despite the rapid loss of LPM in response to i.p. stimuli, it is expected that these cells are retained in the tissue for an immunological purpose. In fact, unpublished data from our group demonstrated their marked ingestion of apoptotic cells followed by IL-10 secretion, which suggest they have some early sentinel role in PerC. Since LPM disappearance is accompanied by the enrichment of SPM and monocytes, the PerC appears to be repopulated with other myeloid subsets that are more competent in disturbed milieus.

LPM, SPM and monocyte frequencies found in stimulated PerC were influenced by the nature of the stimuli (Fig. 2). PC from zymosan-stimulated mice exhibited Ly-6C^+^ monocytes as the dominant population, whereas SPM was the major myelo-monocytic subset present in the PerC of *T. cruzi*-stimulated mice. Since infiltrating SPM and monocytes display the same expression of F4/80, these cells were distinguished by Ly-6C expression found only in monocytes as well as the high levels of MHCII expressed by SPM (Fig. 2). Infiltrated monocytes in PerC seem to display reduced Ly-6C expression when compared to blood monocytes [1], suggesting that these cells differentiate in PerC. Therefore, it is not certain whether infiltrating SPM and monocytes represent two distinct populations or, conversely they are the same population in different maturation states. In our previous study, we found that Ly-6C^+^ monocytes infiltrate the PerC in response to thyoglicolate injection and subsequently up-regulate MHCII expression resembling SPM [1]. As Ly-6C^+^ monocytes are believed to give rise to inflammatory MØ [36], the putative origin of SPM could explain the activated profile exhibited by SPM (Table 1 and Fig. 4).

Of note, independent of stimuli and SPM or monocytes dominancy, stimulated-PC appear to be more easily responsive to subsequent *in vitro* LPS stimulation as they secreted higher levels of NO and exhibited higher frequencies of IL-12-positive cells. Finally, SPM rather than LPM or monocytes, appear to be the major source of IL-12 in response to a subsequent LPS plus IFN-γ stimulation. Moreover, exposure to infectious stimuli led to the emergence of a robust SPM population exhibiting a higher frequency of IL-12-positive cells in response to subsequent LPS plus IFN-γ stimulation when compared to SPM from untreated mice (Fig. 4). Since IL-12 has been described as an important element for driving the polarization of peritoneal MØ towards M1 activation profile and the consequent capacity of these cells to produce NO in response to LPS and IFN-γ [26]–[30], we might propose that in inflammatory conditions PerC is enriched with a primed and easily activated MØ population, which could improve microbicidal responses within this environment.

Thus, the substitution of LPM by SPM and monocytes greatly improves local cell responses to subsequent stimuli. However, lack of experimental models with conditional depletion of MØ subsets prevents us to determine the relative roles of LPM, SPM and monocytes for host protection against infections. Taken together, our data suggest that the infectious stimuli induce the disappearance of senescent LPM, the infiltration of monocytes and the emergence of a robust SPM subset; which collectively provides cellular renewal and lead to an improvement in the PerC response to a subsequent LPS and IFN-γ stimulation.

## Supporting Information

Figure S1 **SSC profile discriminates granulocytes and peritoneal MØ subsets**. (**A**) Granulocytes present a particular SSC profile (Gray dots, total PC; Black dots, indicated F4/80^+^ population), which were used for their exclusion in sequential analysis. (**B**) Plots show the sequential gates used for the exclusion of granulocytes and the subsequent identification of LPM and SPM. Experiments were repeated 3 times, showing similar profiles.
(TIF)
