# Supplementary figures and images for: Cellular Renewal and Improvement of Local Cell Effector Activity in Peritoneal Cavity in Response to Infectious Stimuli
Source: PLoS One. 2011 Jul 22;6(7):e22141. doi: 10.1371/journal.pone.0022141 (PMC3142143; doi:10.1371/journal.pone.0022141)

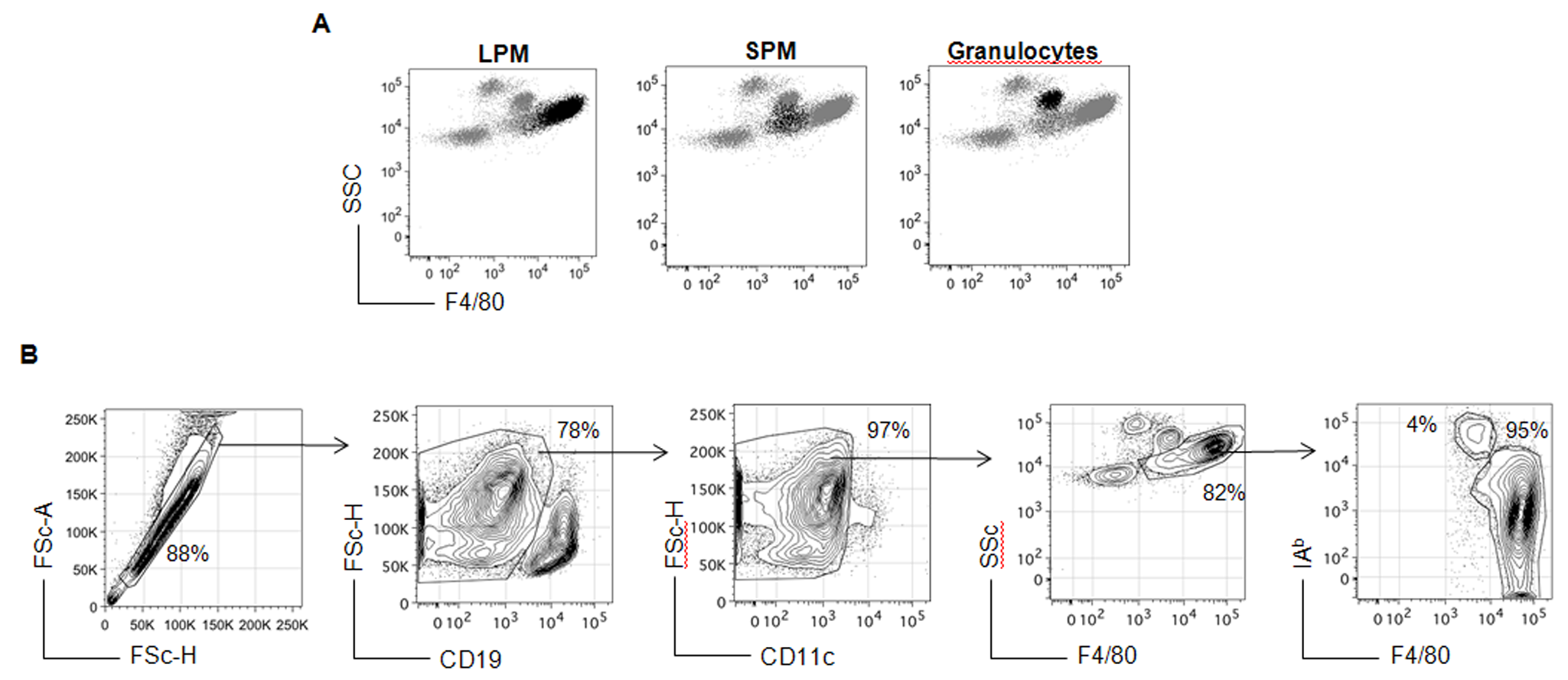

Supplement: Figure S1 — SSC profile discriminates granulocytes and peritoneal MØ subsets. (A) Granulocytes present a particular SSC profile (Gray dots, total PC; Black dots, indicated F4/80+ population), which were used for their exclusion in sequential analysis. (B) Plots show the sequential gates used for the exclusion of granulocytes and the subsequent identification of LPM and SPM. Experiments were repeated 3 times, showing similar profiles. (TIF) [file pone.0022141.s001.tif]
